# The Centrosomal Linker and Microtubules Provide Dual Levels of Spatial Coordination of Centrosomes

**DOI:** 10.1371/journal.pgen.1005243

**Published:** 2015-05-22

**Authors:** Marko Panic, Shoji Hata, Annett Neuner, Elmar Schiebel

**Affiliations:** Zentrum für Molekulare Biologie der Universität Heidelberg, DKFZ-ZMBH Allianz, Heidelberg, Germany; Washington University School of Medicine, UNITED STATES

## Abstract

The centrosome is the principal microtubule organizing center in most animal cells. It consists of a pair of centrioles surrounded by pericentriolar material. The centrosome, like DNA, duplicates exactly once per cell cycle. During interphase duplicated centrosomes remain closely linked by a proteinaceous linker. This centrosomal linker is composed of rootletin filaments that are anchored to the centrioles via the protein C-Nap1. At the onset of mitosis the linker is dissolved by Nek2A kinase to support the formation of the bipolar mitotic spindle. The importance of the centrosomal linker for cell function during interphase awaits characterization. Here we assessed the phenotype of human RPE1 C-Nap1 knockout (KO) cells. The absence of the linker led to a modest increase in the average centrosome separation from 1 to 2.5 μm. This small impact on the degree of separation is indicative of a second level of spatial organization of centrosomes. Microtubule depolymerisation or stabilization in C-Nap1 KO cells dramatically increased the inter-centrosomal separation (> 8 μm). Thus, microtubules position centrosomes relatively close to one another in the absence of linker function. C-Nap1 KO cells had a Golgi organization defect with a two-fold expansion of the area occupied by the Golgi. When the centrosomes of C-Nap1 KO cells showed considerable separation, two spatially distinct Golgi stacks could be observed. Furthermore, migration of C-Nap1 KO cells was slower than their wild type RPE1 counterparts. These data show that the spatial organization of centrosomes is modulated by a combination of centrosomal cohesion and microtubule forces. Furthermore a modest increase in centrosome separation has major impact on Golgi organization and cell migration.

## Author Summary

During most of interphase, the two centrosomes of a cell are kept together by a proteinaceous linker, called the centrosomal linker. It is clear that the linker has to be dissolved by Nek2 kinase and other mechanisms before mitosis in order to assemble a functional bipolar mitotic spindle. Yet the relevance of the centrosome linker for cell function during interphase is not understood. Here we describe for the first time the analysis of a knockout (KO) cell line that lacks an essential component of the centrosome linker, C-Nap1. We observed that centrosomes in these cells are devoid of linker proteins and Nek2 kinase whereas other centrosomal proteins localize to centrosomes as in wild type cells. On average the centrosome distance is moderately increased in C-Nap1 KO cells from 1 to 2.5 μm. We further show that the centrosomal linker is only one element that positions centrosomes close to each other in interphase cells. In linker deficient cells, microtubules spatially organize centrosomes. This resolves a long discussed issue on the role of microtubules in centrosome cohesion. Moreover, we observed that linker deficient cells mis-organize the Golgi. Furthermore, migration of C-Nap1 KO cells was slower than their wild type RPE1 counterparts.

## Introduction

The centrosome is the principal microtubule organizing center (MTOC) in most animal cells. By nucleating and anchoring microtubules, the centrosome influences microtubule directed processes including shape, polarity, organelle transport, adhesion, motility and division of cells [1]. Centrosomes consist of the centrioles and the pericentriolar material (PCM) that has microtubule nucleation activity [2].

In telophase/G1 the two perpendicularly joined centrioles become separated by the activities of polo kinase and separase [3,4]. Simultaneously, a proteinaceous linker, called the centrosomal linker, assembles at the proximal end of the two centrioles and keeps them connected [5]. In G1/S phase, each of the two linked centrioles initiate the process of duplication at the end of which the cell has two centrosomes each with two centrioles. The two centrosomes remain connected by the centrosomal linker [6] until the onset of mitosis when the centrosomal linker is dissolved [7–9]. This enables the two centrosomes to organize the poles of the mitotic spindle and to segregate the chromosomes. Since the two centrosomes are closely connected in interphase by the centrosomal linker, it was suggested that they function as a single MTOC [7].

At the molecular level, several proteins have been shown to play a role in the assembly and disassembly of the centrosomal linker. C-Nap1 acts as a docking site for all linker proteins at the proximal end of centrioles [7,10–14]. The protein rootletin forms filaments that physically connect the two centrosomes [14,15]. Recently, Cep68, LRRC45 and centlein were identified as structural components of the centrosomal linker [11–13]. At the onset of mitosis, enhanced activity of polo kinase Plk1, a major mitotic kinase, activates Nek2A through the Ste20-like kinase Mst2 that directs Nek2A to centrosomes [16,17]. Epidermal growth factor (EGF) also recruits Nek2A to centrosomes and so regulates linker dissolution in a mode of control that is linked to external cues [18]. In addition, cyclin B2 overexpression and p53 transcriptional activity split centrosomes prematurely by activating the Plk1-Mst2-Nek2A pathway [19].

At centrosomes, Nek2A phosphorylates C-Nap1, rootletin and other linker components [7,11,15]. This phosphorylation leads to linker disassembly without degradation of its components. In contrast, phosphorylation of Cep68 by Plk1 in prometaphase triggers proteolytic degradation of Cep68 by the E3 enzyme βTrCP and the proteasome [20]. Cep68 degradation seems to be mainly important for removal of the protein CEP215/CDK5RAP2 from the PCM but not for linker dissolution. After the linker is dissolved, the centrosomes migrate away from one another as a consequence of anti-parallel microtubule sliding forces of the kinesin-5 motor protein Eg5/Kif11 and HKlp2/Kif15 [21–23].

Most studies to date focused on identifying components that are important for centrosomal linker structure and regulation, yet the function of the centrosomal linker remains elusive. Here, we have constructed RPE1 linker deficient cells by inserting a premature stop codon together with the neomycin resistance gene as a selection marker into exon 15 of CEP250 gene (coding for C-Nap1). These C-Nap1 knockout (KO) cells were used to assess the functional importance of the centrosomal linker at the cellular level. C-Nap1 KO cells exhibited normal chromosome segregation and surprisingly the average distance between centrosomes was only modestly increased, suggesting an additional level of centrosomal organization. Microtubules but not dynein or actin were an additional organizing component of inter-centrosome positioning. Nevertheless, the moderate increase in centrosome distance in C-Nap1 KO cells was sufficient to affect Golgi organization and reduce the speed of cell migration.

## Results

### Generation of RPE1 C-Nap1 KO Cell Lines

**Fig 1:**
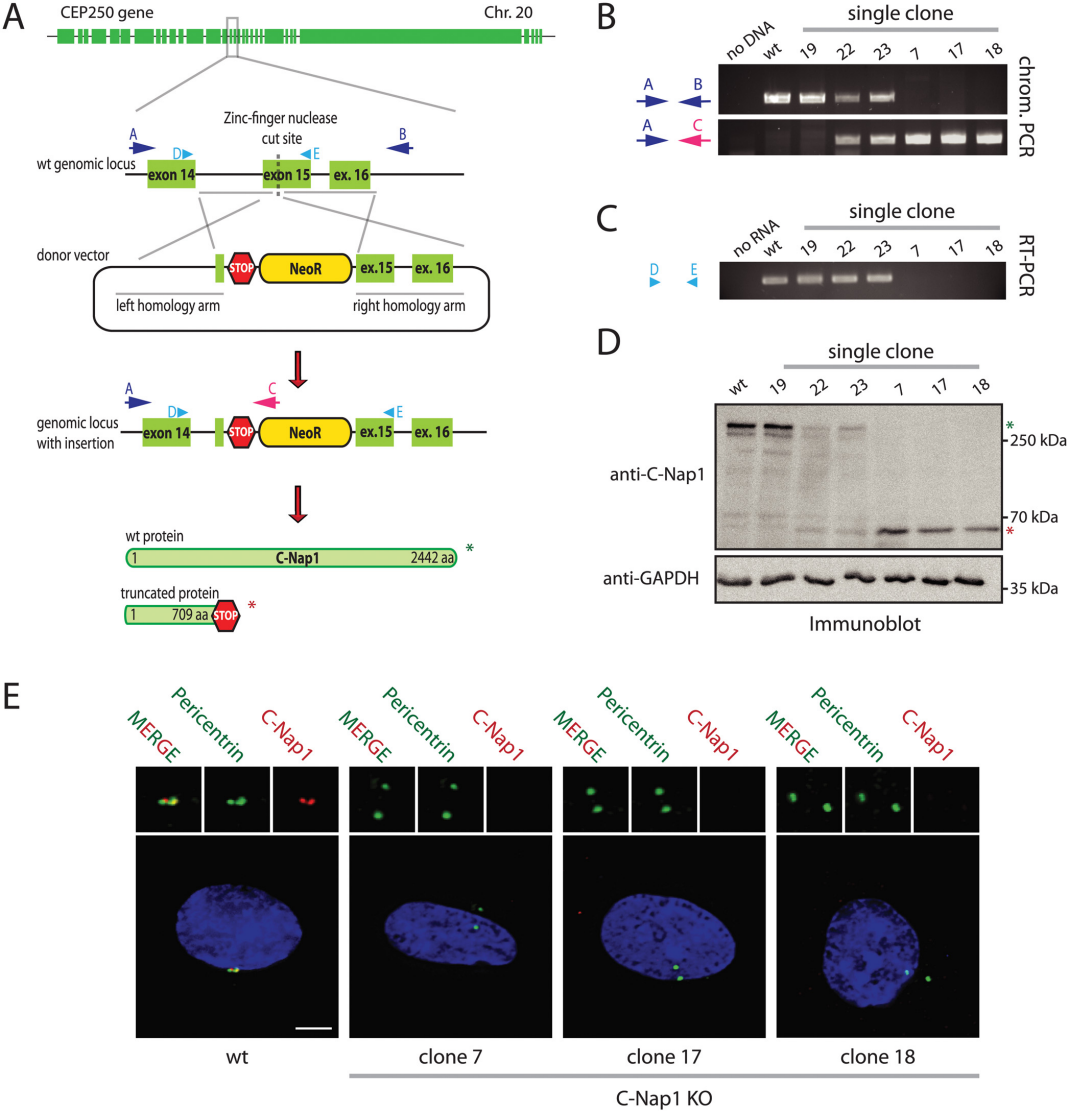
Construction of RPE1 C-Nap1 KO cells. (A) Schematic representation of the CEP250 knockout strategy using a ZFN targeting exon 15 and a neomycin resistance (NeoR) donor construct. Emphasis was put on the exons of the gene. (B) Analysis of clonal RPE1 KO cells by PCR using the primers indicated in (A). The A-C primer pair only yielded a PCR product when NeoR is correctly inserted into CEP250. Primers A and B targeted the whole genomic locus outside of the homology arms. Clones with insertion of NeoR into both CEP250 alleles did not give a PCR product because of the increased length due to the insertion. The ZFN strategy produced rare random integrants (clone 19), but mostly single (clone 22 and 23) and double CEP250 neomycin integrants that targeted both copies of exon 15 (named C-Nap1 KO cells) (clones 7, 17 and 18). (C) RT-PCR of single clones indicating the presence of wt CEP250 mRNA. Primer pair D and E was designed to amplify from exon 14 to the end of exon 15. In case of NeoR integration into exon 15 of CEP250, we did not obtain a PCR product because of the large size. (D) Immunoblots of cell extracts of the indicated clonal cell lines with anti-C-Nap1 antibodies directed against the N-terminus C-Nap1 and anti-GAPDH antibodies. The latter was used as loading control. Single allele knockout clones 22 and 23 have reduced levels of full-length C-Nap1 (green asterisk). We did not detect full-length C-Nap1 in double allele knockout clones 7, 17, and 18. In these clones we observed a truncated version of C-Nap1 (red asterisk) as predicted by the knockout strategy. (E) Immunofluorescence analysis using antibodies directed against the N-terminus of C-Nap1 showed that the truncated C-Nap1 protein does not localize to the centrosome in contrast to the full-length C-Nap1 in wt cells. Bars: 5 μm.

The importance of the centrosomal linker that connects the two centrosomes during interphase is not understood. To gain insights into the cellular function of this connection, we disrupted both copies of the CEP250 gene (encoding for C-Nap1) in hTERT-immortalized retinal pigment epithelial cells (RPE1). C-Nap1 was chosen because all known linker proteins are dependent on this protein for centriole binding [7,10–14]. We employed a zinc finger nuclease (ZFN)-induced homologous recombination strategy in combination with a neomycin resistance donor construct to insert a premature stop codon into the C-Nap1 open reading frame (Fig 1A). The ZFN strategy produced rare random integrants (Fig 1B, clone 19), but mostly single (clone 22 and 23) and double CEP250 neomycin integrants that targeted both copies of exon 15 (named C-Nap1 KO cells) (Fig 1B; clones 7, 17 and 18 are independent clonal cell lines). RT-PCR analysis confirmed the absence of wild type (wt) C-Nap1 mRNA in clones 7, 17, and 18 (Fig 1C). At the protein level, we analyzed C-Nap1 KO clones by immunobloting (Fig 1D). We could observe that the single allele knockout clones 22 and 23 expressed full length C-Nap1 at lower levels compared to the RPE1 wt cell line. Furthermore, full length C-Nap1 was undetectable in the C-Nap1 KO clones 7, 17, and 18. However, the C-Nap1 antibody that is directed against the N-terminus of the protein detected the predicted N-terminal C-Nap1 fragment of 65 kDa in the single (Fig 1D; clones 22 and 23) and double allele knockout cell lines (clones 7, 17, and 18). Using the same C-Nap1 antibody, the full-length protein was detected by indirect immunofluorescence at centrosomes of RPE1 wt cells. Yet we did not observe a centrosomal or another defined signal in the C-Nap1 KO clones 7, 17, and 18 (Fig 1E). Thus, the N-terminal C-Nap1 fragment of C-Nap1 KO cells was unable to provide centrosomal linker function and was most likely dispersed in the cytoplasm. This analysis confirmed that our strategy has generated cell lines that lack functional C-Nap1 protein. Clones 7, 17, and 18 were used in further experiments to address the function of C-Nap1 and the overall function of the centrosomal linker.

### Centrosomes of RPE1 C-Nap1 KO Cells Lack Centrosomal Linker Proteins, yet Remain in Close Proximity

**Fig 2:**
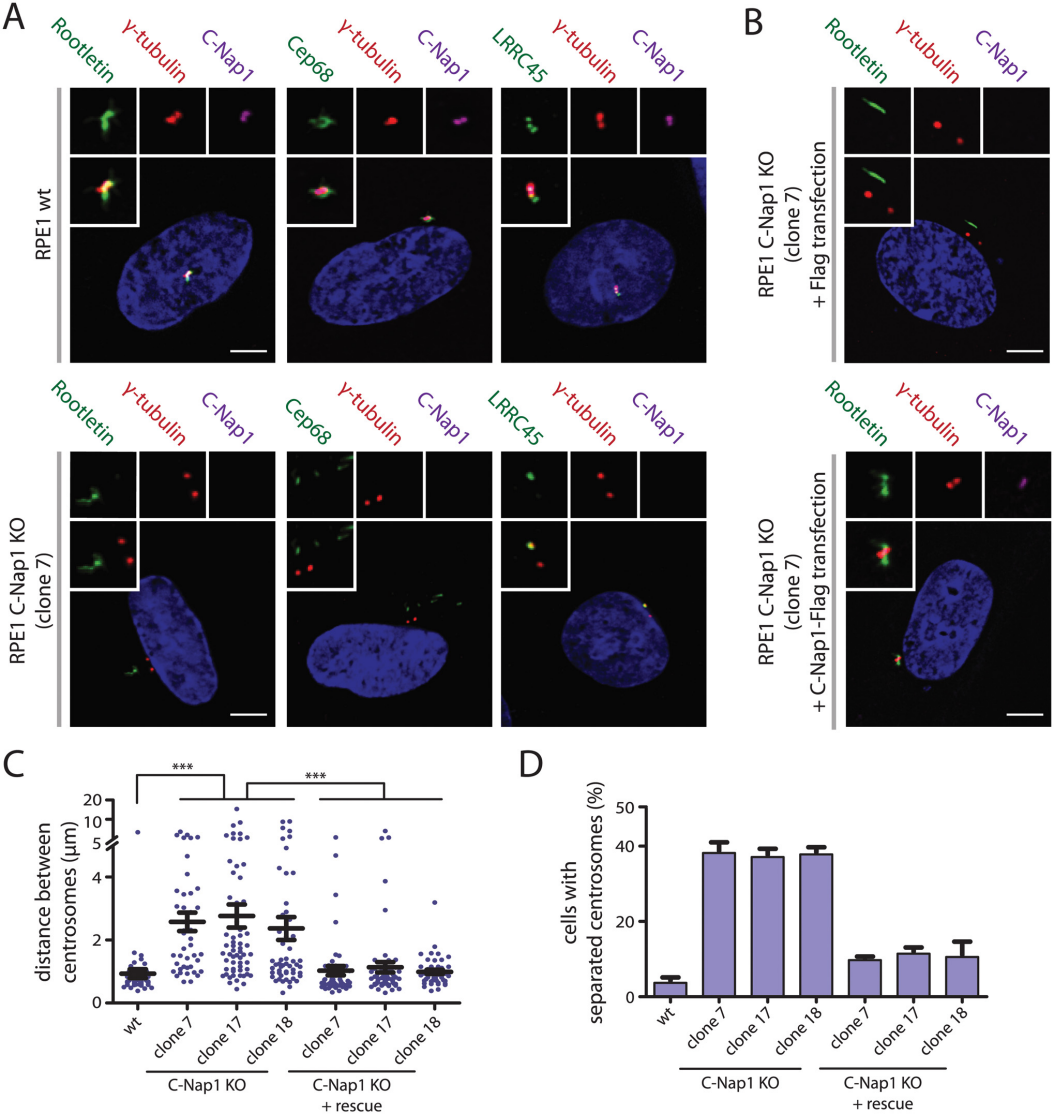
Centrosomes of RPE1 C-Nap1 KO cells lack a centrosomal linker, yet they are kept relatively close together. (A) RPE1 wt and RPE1 C-Nap1 KO cells were fixed and stained with the indicated linker specific antibodies. DNA was stained with DAPI. Three-fold magnifications of centrosomes are shown above. Note, linker proteins were no longer associated with centrosomes of C-Nap1 KO cells. Bars: 5 μm. (B) Complementation of the linker defect in RPE1 C-Nap1-KO cell lines by transient C-Nap1 expression. Cells were fixed and analyzed with the indicated antibodies. Note, strong C-Nap1 overexpression resulted in C-Nap1 aggregates in the cytoplasm. We picked cells with close to physiological C-Nap1 expression levels. Bars: 5 μm. (C) Average distance of centrosomes in fixed RPE1 wt and several independent clonal RPE1 C-Nap1 KO cell lines. C-Nap1 KO cells show an increase of average centrosome distance from 1 to 2.5 μm. N = 40–60 cells were analyzed per cell line. *** p<0.001. Error bars are SEM around the mean value. (D) Cells of (C) were categorized according to centrosome distance. Centrosome distance of >2 μm was taken as “separated centrosomes”. N = 50 cells were analyzed per cell line. Error bars represent SEM of 3 independent experiments.

siRNA depletion of C-Nap1 impairs localization of all other linker proteins [12]. Consistent with these data, rootletin and Cep68 were no longer associated with centrosomes in two independent C-Nap1 KO cell lines (Fig 2A, S1A and S1B Fig). However, we noticed cytoplasmic filament-like assemblies of rootletin and Cep68 in 20–30% of C-Nap1 KO cells that were not connected to centrosomes and did not contain the N-terminal C-Nap1 fragment (Fig 2A and S1B Fig). This suggests that C-Nap1 is not needed as an organizer for rootletin/Cep68 filaments per se, rather for the anchorage of these filaments to centrosomes.

LRRC45 that associates with the linker and with appendages of the mother centriole [12], lost linker localization in C-Nap1 KO cells but still bound to appendages (Fig 2A and S1B Fig). Nek2A no longer associated with the centrosomes of C-Nap1 KO cells indicating that the majority of this kinase binds to centrosomes via linker proteins (S1A and S1B Fig) [24]. Interestingly, Nek2A did not colocalize with the rootletin or Cep68 filaments that were observed in the proximity of centrosomes in C-Nap1 KO cells (S1A and S1B Fig). Transient transfection of C-Nap1 KO cells with a C-Nap1 expression construct restored localization of centrosomal linker proteins and centrosomal linker function (Fig 2B–2D).

Analysis of other centrosomal proteins revealed no change in the distribution of centrin [25], the centriole duplication proteins Cep135 and Sas-6 [26,27], the PCM proteins pericentrin and γ-tubulin [28,29], and the distal appendage protein Cep164 [30] (S1A and S1B Fig). Thus, although centrosomes from C-Nap1 KO cells lack centrosomal linker proteins, the localization of other centrosomal proteins was as in wt cells. Consistent with this conclusion, analysis of C-Nap1 KO cells by electron microscopy did not reveal obvious changes in centriole structure when compared to RPE1 wt cells (S2 Fig).

The term pericentriolar satellite defines electron-dense granules around the centrosome. These granules recruit centrosomal proteins and have functions in cilia formation [31,32]. In C-Nap1 KO cells the pericentriolar satellite protein PCM-1 [33] was preferentially positioned towards the Cep164-marked mother centriole (S1A–S1C Fig). Because of the close linkage of both centrioles in RPE1 wt cells, it was impossible to say whether this was the normal behaviour of pericentriolar satellites. The relevance of this asymmetric localization of PCM-1 remains unclear but it did not lead to a cilia formation defect in RPE1 C-Nap1 KO cells (S3A and S3B Fig), as this has been reported for siRNA depletion of pericentriolar satellite proteins [32,34]. Cilia formation in C-Nap1 KO cells is consistent with the published assembly of cilia upon siRNA depletion of C-Nap1 [30].

One of the main functions of centrosomes is the formation of the mitotic spindle. Analysis of mitotic C-Nap1 KO cells did not reveal any striking mitotic defects such as lagging or mis-segregated chromosomes (S4 Fig). The bipolar mitotic spindles of C-Nap1 KO cells (N = 20) were normal in appearance, suggesting that the main cellular function of the centrosomal linker is not in mitotic spindle formation.

### Microtubules Position Centrosomes in C-Nap1 KO Cells in Relatively Close Proximity to Each Other

Interfering with linker proteins increases the distance between both centrosomes during interphase [10–13]. We also observed an increase in average inter-centrosome distance from 1 μm in RPE1 wild type cells to 2.5 μm in three independent C-Nap1 KO cell lines (Fig 2C). When we categorized cells with a centrosome distance >2 μm as separated, only ~35% of C-Nap1 KO cells had separated centrosomes compared to the 5% recorded for wt cells (Fig 2D). These findings suggest that in the absence of the centrosomal linker another mechanism keeps the two centrosomes near to one another.

**Fig 3:**
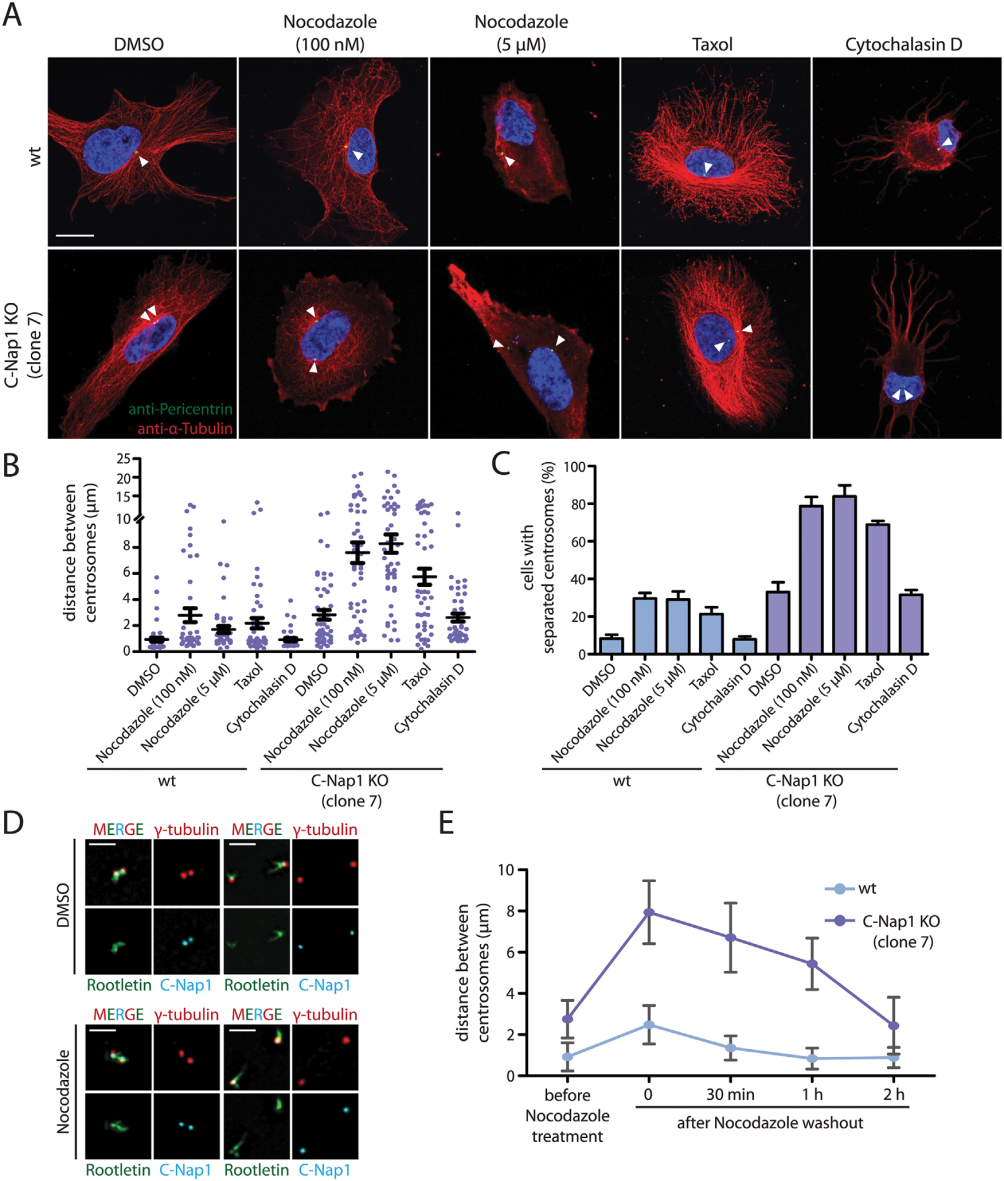
In the absence of a centrosomal linker, microtubules position the two centrosomes of a cell relatively close together. (A) RPE1 wt and RPE1 C-Nap1 KO cells were treated with the indicated reagents for 1 h at 37°C. Fixed cells were analyzed by indirect immunofluorescence with the indicated antibodies. Size bar: 10 μm. (B) Distance between centrosomes of RPE1 C-Nap1 KO cells increases upon treatment of cells with the microtubule drugs nocodazole and taxol. Cells were incubated with the indicated drugs for 1 h at 37°C and subsequently fixed and stained with γ-tubulin antibodies. The distance between centrosomes was determined as described in Materials and Methods. N = 40–60 per condition. Error bars are SEM around the mean value. (C) Cells from (B) were grouped into “cells with separated centrosomes” when the centrosomal distance was >2 μm. Error bars are SEM between 3 independent experiments. (D) Linker morphology in RPE1 cells. RPE1 wt cells were incubated for 1 h with 5 μM nocodazole or the solvent control DMSO. Cells were fixed and stained with the indicated linker antibodies and the centrosome marker γ-tubulin. RPE1 wt cells with a centrosome distance <2 μm have a functional centrosomal linker. Centrosomes of cells with a distance >2 μm are associated with linker proteins but the connection is not established. Nocodazole treatment does not cause displacement of C-Nap1 and rootletin from centrosomes. Bar: 2 μm. (E) The effect of nocodazole on the centrosome distance of RPE1 C-Nap1 KO cells is reversible. Cells were treated for 1 h with 100 nM nocodazole. Nocodazole was washed out (t = 0) the centrosome distance in fixed cells was determined 30, 60 and 120 min after wash out. N = 50 cells per time point and cell line. Error bars are SEM.

Early studies have shown that treatment of cells with nocodazole promotes centrosome separation [35,36]. This observation has been interpreted in different ways (see Discussion), however, the authors of these studies were not in a position to analyze the role of microtubules in centrosome positioning in the absence of centrosomal linker function. To this end, we perturbed microtubule and actin function with nocodazole, taxol and cytochalasin D, respectively. Nocodazole at 5 μM completely depolymerised microtubules (Fig 3A). In C-Nap1 KO cells, nocodazole drastically increased the average distance between centrosomes from 2.5 μm to ~8 μm (Fig 3B). Centrosome separation increased from 35% to ~80% upon drug treatment (Fig 3C).

In contrast, in RPE1 wt cells 5 μM nocodazole only moderately increased average centrosome distance from 1 to 2.5 μm (Fig 3B). It is important to note that these cells fell into two phenotypic groups. 70% of RPE1 wt cells maintained the short centrosome distance of 1 μm. In 30% of cells, centrosome distance increased to 4–15 μm, indicative of a failure of centrosomal linker function (Fig 3B). In order to understand why short treatment with nocodazole increases centrosome separation only in some interphase cells, we analyzed the state of the centrosomal linker of RPE1 wt cells in the presence and absence of nocodazole. RPE1 centrosomes associated with the linker proteins C-Nap1 and rootletin independent of their distance and whether cells were treated with nocodazole (Fig 3D). However, while centrosomes with a distance <2 μm were connected by rootletin fibres (Fig 3D, cells on the left), centrosomes with a distance >2 μm carried two unconnected rootletin bundles (Fig 3D, cells on the right). This was observed invariantly of nocodazole treatment. Thus, separated interphase centrosomes of RPE1 wt cells carry linker proteins but these are disconnected and therefore non-functional.

We next asked whether microtubule dynamics is important for the centrosome position in linker deficient cells. Low concentrations of nocodazole (100 nM), had only a modest impact on the integrity of the microtubule cytoskeleton (Fig 3A). At this concentration nocodazole mainly affects microtubule dynamics rather than overall architecture [37]. Despite the presence of a microtubule network, 100 nM nocodazole was as efficient in inducing centrosome separation in RPE1 C-Nap1 KO cells as complete microtubule depolymerization (Fig 3B and 3C). Interestingly, the effect of microtubule depolymerisation on centrosome positioning in C-Nap1 KO cells was reversible. Nocodazole wash out restored the relatively close juxtaposition of centrosomes within 2 h (Fig 3E). Thus, changes in microtubule dynamics are probably sufficient in disturbing centrosome positioning. Consistent with this notion, we observed that the microtubule stabilizer taxol also increased the distance between unlinked centrosomes, while having only a minor impact on centrosomes separation in RPE1 wt cells (Fig 3B and 3C).

Cells treated with cytochalasin D showed a complete collapse of the actin cytoskeleton (S5 Fig). However, actin depolymerization did not induce centrosome separation of C-Nap1 KO cells (Fig 3A–3C). Thus, we concluded that microtubules, not actin, maintain the proximity of unlinked centrosomes.

The microtubule motor protein dynein positions centrosomes to defined cellular locations in a number of cell types [38–40]. We therefore asked whether dynein is important for centrosome coordination in linker deficient C-Nap1 KO cells. Disruption of dynein motor activity using the dynein inhibitor ciliobrevin D [41] did not increase the distance between centrosomes in C-Nap1 clones (S6A and S6B Fig). The same was observed in overexpression experiments with the dynein inhibitor construct p50/dynamitin [42] (S6C and S6D Fig). Disorganization of the Golgi network was used as control for dynein inhibition in both experiments (S6A and S6C Fig). Thus, the spatial coordination of centrosomes in C-Nap1 KO cells is not dependent on dynein and likely requires the activity of other microtubule and/or cell cortex associated proteins.

### Dynamics of Centrosomes in C-Nap1 KO Cells

**Fig 4:**
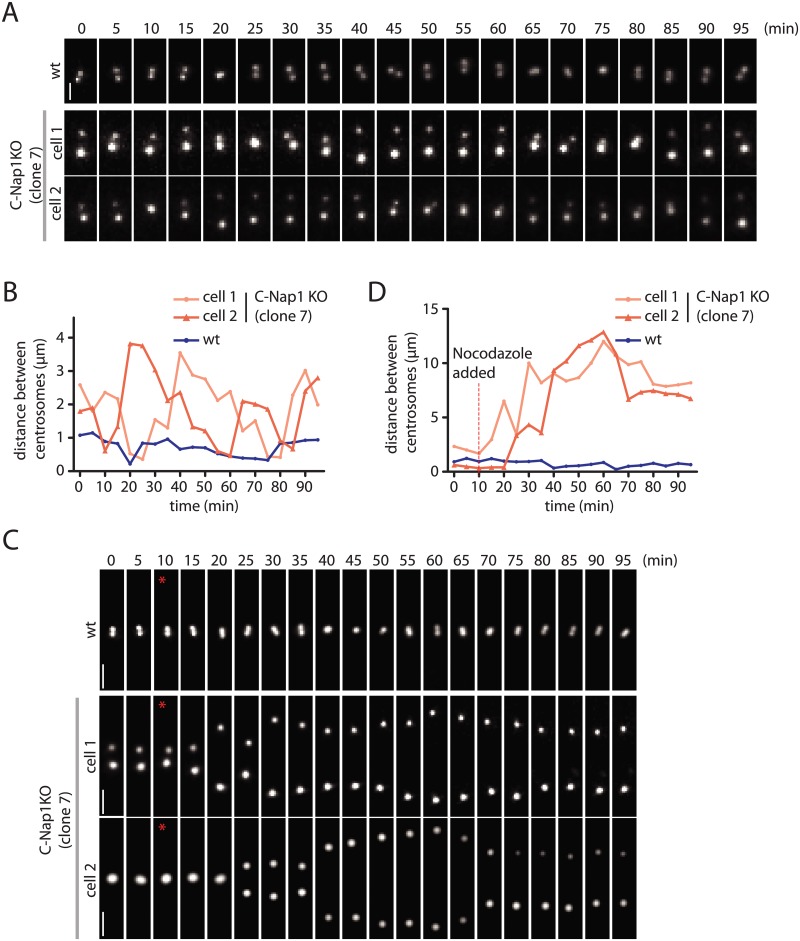
Live cell imaging of centrosome behaviour in RPE1 wt and RPE1 C-Nap1 KO cells. (A) RPE1 wt and RPE1 C-Nap1 KO cells were transfected with NeonGreen-PACT DNA. Centrosome distance was analyzed over time. Size bar: 2 μm. Representative images from 20 analyzed cells are shown. (B) Quantification of (A). Centrosome distance over time. The fluctuation of centrosome distance in the RPE1 C-Nap1 KO cells is clearly greater than in RPE1 wt cells. (C) As in (A), however, 100 nM nocodazole was added 10 min after start of the imaging (red star). Size bar: 3 μm. Representative images from 20 analyzed cells are shown. (D) Quantification of (C). Centrosome distance over time. The red line indicates addition of 100 nM nocodazole. 10–15 min after addition of nocodazole, centrosomes separated in RPE1 C-Nap1 KO cells while they were kept close together by the linker in the majority of RPE1 wt cells.

The data above do not give insights into the dynamics of centrosomes in the absence of linker proteins. We addressed this point by transfecting RPE1 wt and C-Nap1 KO cells with a construct expressing mNeonGreen-PACT. The PACT domain targets the fluorophore to the centrioles [43]. In RPE1 wt mNeonGreen-PACT cells, the distance between the two centrosomes remained relatively constant at 0.5–1.0 μm (Fig 4A and 4B). Time lapse analysis indicated that the two centrosomes of C-Nap1 KO cells moved back and forth but kept an average distance of 2 μm and rarely separated further apart than 3.5 μm (Fig 4A and 4B). In confirmation of the data from fixed cells, 100 nM nocodazole treatment uncoupled the two centrosomes of C-Nap1-KO cells (Fig 4C and 4D). Shortly after nocodazole addition, the two centrosomes separated by up to 10–12 μm and maintained an average distance of 8–9 μm (Fig 4D). Live cell analysis therefore supported the view that in the absence of the centrosomal linker, microtubule dependent forces coordinate the closed spatial positioning of centrosomes.

Based on these experiments, we conclude that in RPE1 cells the centrosomal linker maintains the stable association of centrosomes. In the absence of the linker, microtubule forces keep the two centrosomes relatively close together.

### Centrosomal Linker in U2OS and HeLa cells

The data above showed that centrosomal linker function is not essential for cell viability and spatial centrosome organization in RPE1 cells. In order to provide a coherent picture on centrosome linkage and to understand the contribution of the centrosomal linker and microtubules on centrosome positioning in different cell lines, we analyzed RPE1, Human Bone Osteosarcoma Epithelial Cells (U2OS) and HeLa cells in combination with siRNA depletion of C-Nap1 and nocodazole treatment (S7 Fig). Identical data as for RPE1 C-Nap1 KO cells were obtained upon siRNA depletion of C-Nap1 in RPE1 cells (S7A–S7C Fig) demonstrating that the RPE1 C-Nap1 KO phenotypes were not affected by adaptation or expression of the N-terminal C-Nap1 fragment. Consistent with published data [7], U2OS cells had a robust centrosomal linker and microtubule forces kept centrosomes relatively close together in the absence of linker function (S7D–S7F Fig).

The high genetic instability makes HeLa cells heterogeneous. We therefore analyzed two HeLa cell lines of different origin. Interestingly, the average centrosomal distance in HeLa-ATCC and HeLa-B cells was 2.5 to 4-fold higher than in RPE1 and U2OS cells (S7B, S7E, S7H, and S7K Fig). The basal level of centrosome separation was 35% for HeLa-ATCC and 60% for HeLa-B cells (S7I and S7L Fig). Interestingly, HeLa-ATCC cells fell into two groups. 70% had a centrosome distance of 1–2 μm, the distance of the others was >2 μm. This variation indicates heterogeneity in centrosomal linker function within this cell population. A substantial portion of HeLa-ATCC cells had centrosomal linker function as indicated by the C-Nap1 siRNA depletion induced centrosome separation from 35 to 60% (S7I Fig). Nocodazole treatment and C-Nap1 depletion had a synergistic effect on centrosome separation (S7H and S7I Fig).

HeLa-B cells had very little, if any, centrosomal linker function since C-Nap1 depletion hardly increased the already high level of centrosome separation (S7K and S7L Fig). However, microtubule depolymerization increased the centrosome distance from 4 to 6 μm (S7K Fig) indicating that microtubules provided some centrosome coordination in these cells.

These differences in centrosome behaviour were reflected in the morphology of the linker (S8 Fig). Most HeLa-B cells did not have a connecting centrosomal linker independent of the centrosome distance although linker proteins were associated with centrosomes (S8C Fig). U2OS and HeLa-ATCC cells had a functional linker when the centrosome distance was <2 μm (S8A and S8B Fig). Taken together, centrosomal linker function is variably established in human cell lines.

### The Centrosmal Linker Is Important for Golgi Organization and Cell Migration

**Fig 5:**
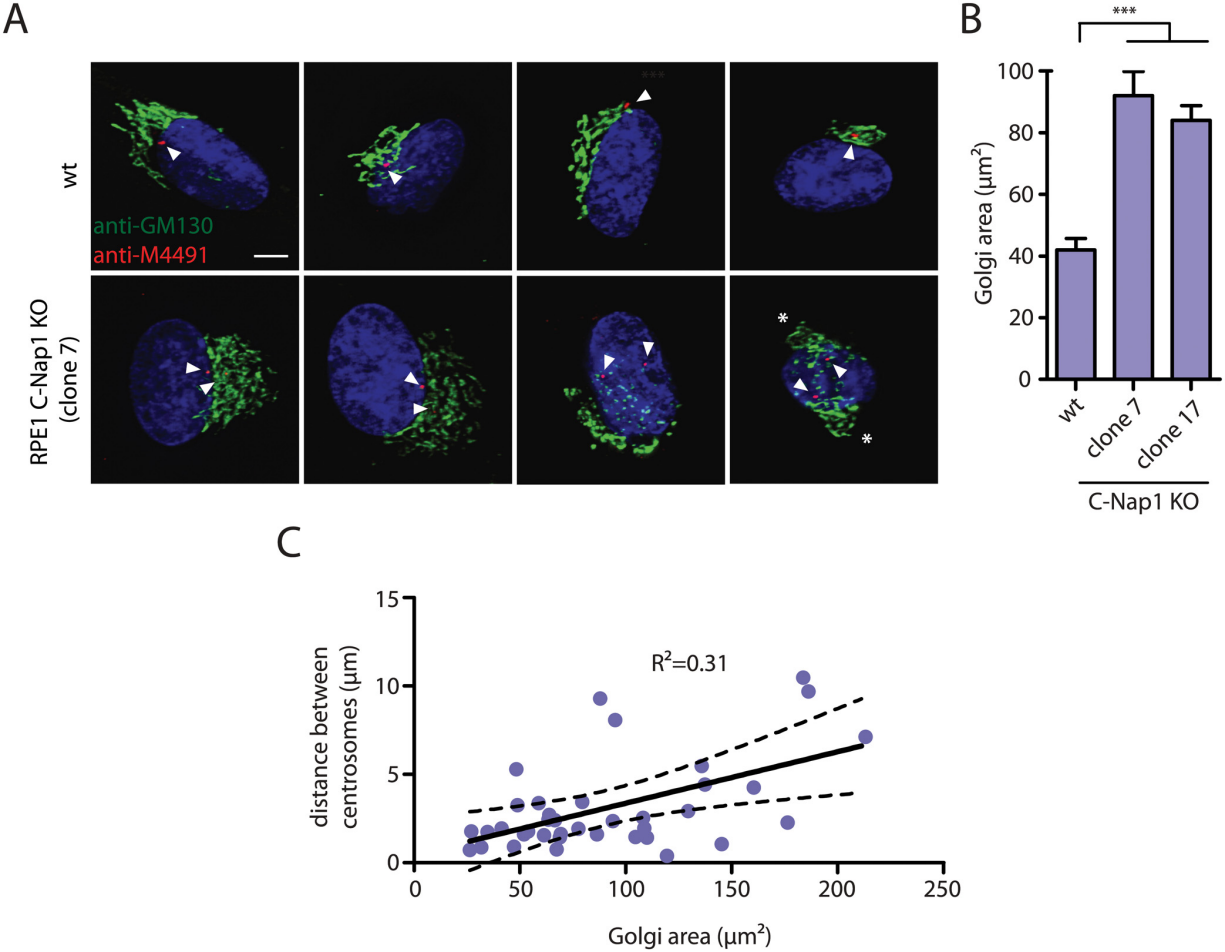
RPE1 C-Nap1 KO cells have Golgi organization defects. (A) Interphase RPE1 wt and RPE1 C-Nap1 KO cells were fixed and stained with the indicated antibodies. DNA was stained with DAPI. Arrowheads mark the position of the two centrosomes of an interphase cell. The white asterisks in the right picture highlight two well separated Golgi stacks. Size bar: 5 μm. (B) The Golgi area that is occupied by cells in (A) was quantified. The Golgi of RPE1 C-Nap1 KO cells occupies a two-fold larger area then the Golgi of RPE1 wt cells. N = 30. Bars are SEM. *** p<0.001. (C) Correlation analysis between Golgi area and centrosome distance. Data from (A) were analyzed as described in Materials and Methods. The dashed lines indicate 95% confidence interval. N = 30.

Recent data suggest a linkage between centrosomes and Golgi function [44,45]. We therefore analyzed distribution of the Golgi marker GM130 in RPE1 wt and C-Nap KO cells (Fig 5A and 5B). The area occupied by the Golgi increased at least 2-fold in C-Nap1 KO cells. In addition, we observed correlation between the centrosome distance and the Golgi area (Fig 5C) suggesting that the increase in centrosome distance is to some extent causing the Golgi organization defect. In cells with a centrosome distance of >8 μm, two well-separated Golgi stacks could even be observed (Fig 5A, cell on the right, white asterisks). Such observations suggest that both of the centrosomes are capable of independently organizing the Golgi stack.

**Fig 6:**
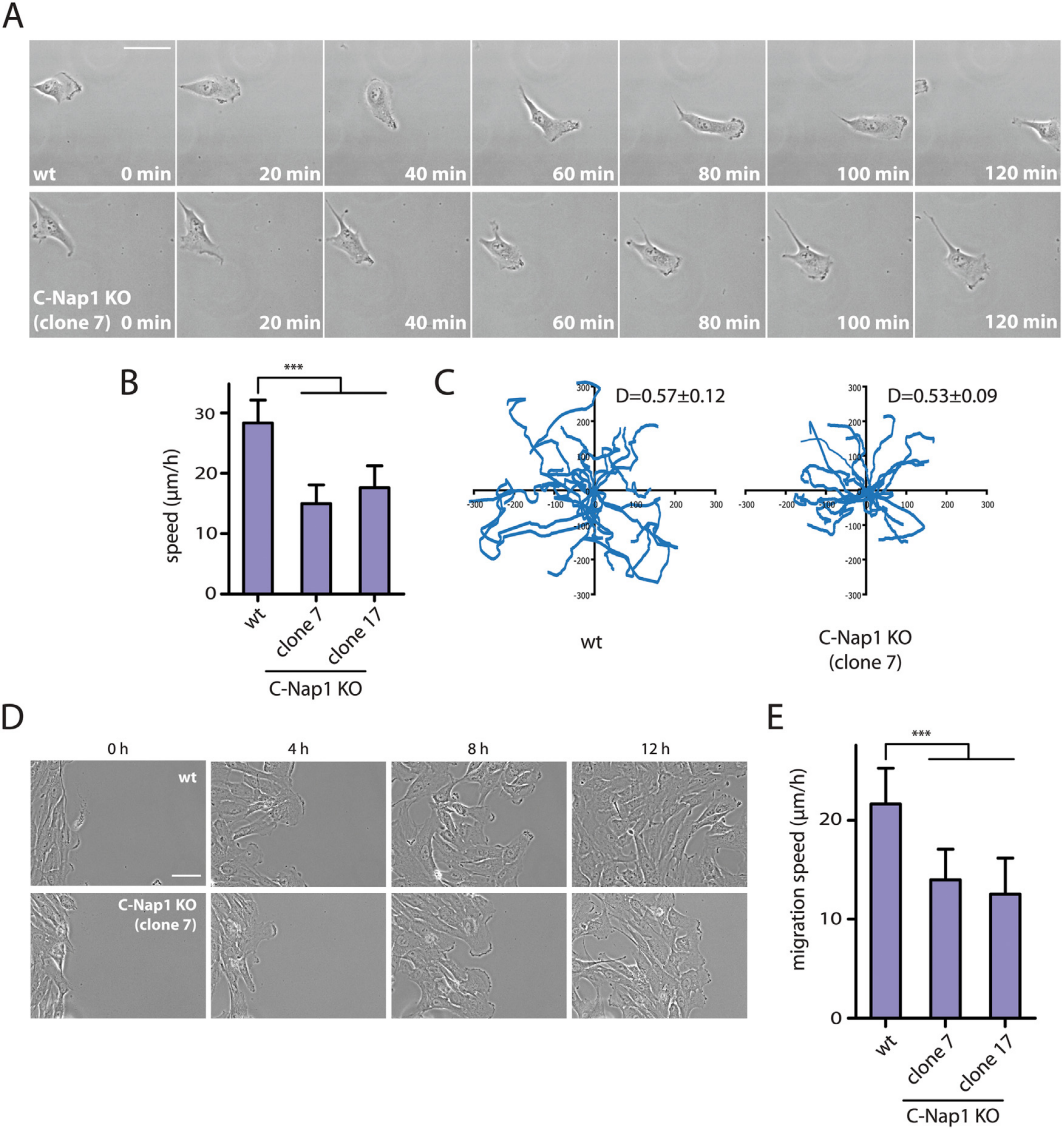
RPE1 C-Nap1 KO cells have reduced migration speed. (A) Analysis of migration speed of RPE1 wt and RPE1 C-Nap1-KO cells. Phase contrast images of migrating cells over time. Bar: 30 μm. (B) Quantification of migration speed of cells from (A). Two independent C-Nap1 KO cell lines were analyzed. RPE1 C-Nap1 KO cells have reduced migration speed compared to RPE1 wt cells. N = 20–30. Bars are SEM. *** p<0.001 (C) Tracking of RPE1 wt and RPE1 C-Nap1 KO cells. The results of the manual cell tracking over a period of 10 h are shown (interval time—15 min). RPE1 wt and RPE1 C-Nap1 KO cells did not show a difference in migration directionality. Values of the directionality index (D±SEM) are shown at the upper right corner of the graph. (D) The wound healing assay showed that C-Nap1 KO clones have a reduced migration speed. Bar 50 μm. (E) Quantification of (D) shows that C-Nap1 KO clones 7 and 17 have reduced migration speed compared to the RPE1 wt control, but to a slightly lesser extent in comparison to the random migration results from (A). Bars are SEM. N = 25–30, *** p<0.001.

An increase in centrosome number affects the migration behaviour of cells [46,47]. We therefore tested whether the increase in centrosome distance in RPE1 C-Nap1 KO cells, also has consequences on cell migration speed. Time-lapse analysis measured a medium speed of 28 μm/sec of RPE1 wt cells. Strikingly, the two independent C-Nap1 KO cells moved at a markedly reduced rate of only 15 and 18 μm/sec, respectively (Fig 6A and 6B). Using the random migration data, we analyzed if the directionality of movement was also altered in C-Nap1 KO cells. However, no significant difference was observed between the C-Nap1 KO and wt RPE1 cells as indicated by the very similar directionality index which is calculated as a ratio between Euclidean and accumulated distance.

A wound healing assay was performed in order to test if directed cell migration was also affected. RPE1 C-Nap1 KO cells also had a reduced migration speed in this assay compared to wt cells, but to a lesser extent than in the random migration assay (Fig 6D and 6E). Both migration assays confirm that unlinked centrosomes are reducing the speed of cell migration.

## Discussion

For the majority of interphase, a proteinaceous linker, called the centrosomal linker, physically connects the two centrosomes of a cell close together. The linker needs to be dissolved by Nek2A kinase and other mechanisms at the onset of mitosis in order to allow the assembly of a bipolar mitotic spindle [7,9,16,18,19]. However, the relevance of centrosome linkage for cell function during interphase is not understood, largely due to the lack of a model cell line in which the linker function is impaired.

C-Nap1 is the central anchor point of linker proteins at the proximal end of the two connected centrioles and its absence impairs binding of all other linker proteins [7,10–15]. RPE1 cells, in which both CEP250 copies were disrupted and hence lack the centrosomal linker, (Figs 1, 2, and S1 Fig) provide an excellent model system for the functional analysis of centrosomal linker function. As predicted from observations of the consequences of interfering with the C-Nap1 function [10], centrosome distance increased from 1 to 2.5 μm in C-Nap1 KO cells. However, to our surprise, some level of spatial organization persisted in the centrosomes of linker deficient interphase cells (Figs 3 and 4). We have determined that the microtubule cytoskeleton plays a role in keeping unlinked centrosomes close together. Low concentration of nocodazole, that mainly affect microtubule dynamics but not microtubule integrity, or the microtubule stabilizer taxol, impaired the positioning of the two unlinked centrosomes and increased the average distance from 2.5 to 8 μm (Fig 3B). We therefore suggest that forces that are dependent on the dynamic properties of microtubules position the two centrosomes in relatively close proximity when the centrosomal linker is missing. Dynein positions centrosomes in other model systems like *C*. *elegans* and *Drosophia* [38,39] but did not affect the distance of centrosomes in linker deficient C-Nap1 KO cells (S6 Fig). Identifying the players that keep centrosomes together in the absence of the linker is an important task for future studies, both from the perspective of centrosome biology in its own right and because this mechanism may contribute to the clustering of over-amplified centrosomes that supports the viability of cancer cells [48].

Previous studies have reported an impact of microtubules on interphase centrosome separation [35,36]. These results were first interpreted as microtubule tension forces that target the two unlinked centrosomes to the same cellular location [35]. With the discovery of the centrosomal linker, the nocodazole induced centrosomal separation was interpreted as a shift in balance between kinases and phosphatases that regulate linker proteins [36]. If the latter model was correct, we should not have observed an increase in centrosome separation in the C-Nap1 KO cell line upon microtubule depolymerization. Furthermore, upon nocodazole induced centrosome separation in RPE1 wt cells, we observed that the centrosomal linker was still at the centrosomes. We propose that both the centrosomal linker and microtubule dependent forces cooperate to keep the centrosomes in close proximity.

Our data suggest that 95% of RPE1 cells have a robust linker that keeps the centrosomes together (Fig 2). FRAP data on centrosomal linker proteins C-Nap1 and rootletin are consistent with this notion of a relatively stable linker [24]. Puzzlingly, despite this linkage, treatment for 1 h with either nocodazole or taxol increased the number of RPE1 cells with disengaged centrosomes from 5 to 20–30% (Fig 3C). We propose that the linker can temporarily loose connection between the two centrosomes with low frequency for example through the loss of rootletin-rootletin interactions within the rootletin polymer [15]. This model is supported by the split linker morphology of RPE1 wt cells with separated centrosomes (Fig 3D). In the absence of coordinating microtubules, centrosomes are able to separate beyond a recoverable distance and the breakage of the linkage becomes permanent. In the presence of microtubules the centrosomes are kept in close proximity to one another and the linker reforms between the two centrosomes to restore centrosome cohesion. Alternatively, cell cycle differences regarding the centrosomal linker may explain the nocodazole induced centrosome splitting. Only cells in a particular cell cycle phase could be more sensitive to centrosomes splitting in response to microtubule depolymerisation while the centrosomal linker of cells in other cell cycle phases remains unaffected.

Currently we do not know the exact mechanism by which microtubules keep unlinked centrosomes in relative close proximity. Here we discuss three models that are not mutually exclusive. The first model includes a possibility that there is another centrosomal linker that is C-Nap1 independent. We do not favour this view since we have shown using live cell imaging that unlinked centrosomes are moving back and forth and do not show the same coordinated movement like their linked counterparts in RPE1 wt cells (Fig 4). In the second model, the two unlinked centrosomes are close together because of similar spatial coordination. It has been suggested that microtubules originating from the two centrosomes interact with dynein at the cell cortex and equal pulling forces position both centrosomes in the cell center [40]. Such a hypothesis would argue that the relative proximity of unlinked centrosome would be dynein dependent. In fact, we have shown that dynein is not responsible for the proximity of unlinked centrosomes (S6 Fig). However, motor proteins other than dynein may position centrosomes in this way in RPE1 cells.

A third possibility is that the microtubules organized by the two centrosomes overlap and are used to establish the close inter-centrosome distance. Motors at centrosomes or associated within the anti-parallel microtubule overlap could position the two centrosomes close to each other. Such principle was shown to be important in yeast karyogamy where the minus end directed motor protein Kar3 is positioned at both spindle pole bodies (SPBs). Kar3 at one SPB pulls on microtubules organized by the other SPB. In this way the two nuclei are moved together until they finally fuse [49]. Additional experimental work is necessary to discriminate between these models.

Using C-Nap1 siRNA depletion and nocodazole treatment, we have analyzed RPE1, U2OS and HeLa cells to obtain a coherent picture of the spatial organization of centrosomes. In RPE1 and U2OS cells, we observed robust linker formation (S7 and S8 Figs). In both cell lines, C-Nap1 depletion and nocodazole treatment showed a clear synergistic effect on centrosome separation indicating that both organizing principles were active in these cells. Efficient centrosomal linker formation in U2OS and RPE1 cells is consistent with published data [7,15] and our RPE1 C-Nap1 KO analysis. Centrosomes of HeLa-ATCC cells responded to C-Nap1 depletion and microtubule depolymerization, indicating that both mechanisms were active. However, the relative high basal level of centrosome separation of 30% suggests that centrosome cohesion was suboptimal in these cells (S7I Fig). In HeLa-B cell the basal level of centrosome separation was very high at 60% (S7L Fig). Two lines of evidence suggest that the linker is not functional in HeLa-B cells. First, in most HeLa-B cells the centrosomal linker was not clearly connecting the two centrosomes even when they were close together (S8C Fig). Second, the high basal level of centrosome separation with an average distance of 4 μm indicates linker defects (S7K Fig). The reason for this failure in linker establishment in HeLa-B cells is presently unclear. High EGF signalling, overexpression of cyclin B2 or the misbalanced expression of linker proteins may cause this defect [18,19]. Furthermore, differences in microtubule dynamics could play a role in explaining the differences between different cell lines.

In any case, our observation of a non-functional linker in some cell lines may help to explain previous experimental findings. For example, siRNA depletion of CDK5RAP2, a microtubule organizing protein that is not associated with the linker [50], increased centrosome distance in U2OS and HeLa cells [13,51]. The penetrance of this phenotype appeared to be stronger in HeLa cells than in U2OS cells. This centrosome separation phenotype may be caused by altered microtubule properties in the cells. In addition, HeLa cells were reported to have highly mobile centrosomes with fluctuating inter-centrosomal distance [52,53]. Having in mind the data presented in this manuscript, a possible interpretation would be that these HeLa cells did not have a functional centrosomal linker allowing centrosome separation.

Recent data suggest a linkage between centrosomes and Golgi function [44,45]. These reports motivated us to analyze Golgi structure in RPE1 C-Nap1 KO cells. The Golgi of C-Nap1 KO RPE1 cells is spread over a wider area than in wild type cells (Fig 5B). A linkage of the Golgi stacks with centrosomes via microtubules is well established [44,45,54]. Microtubules organized by centrosomes interact with Golgi stacks [44]. The Golgi apparatus is also a MTOC that recruits γ-tubulin complexes through the proteins GM130, AKAP450, CDK5Rap2 and myomegalin [44,45,55]. The correlation between centrosome distance and the area covered by the Golgi (Fig 5C) suggests that the increase in centrosome distance is causing the Golgi organization defect. We propose that the increase in centrosome distance in C-Nap1 KO cells leads to greater spreading of the microtubules that are organised by the centrosomes to distribute the Golgi stacks over a larger area. Supporting this notion, when centrosomes of C-Nap1 KO cells were separated by >8 μm, two spatially organized Golgi organelles could be observed (Fig 5B). Such observation suggests that the two unlinked centrosomes can independently organize Golgi stacks. Moreover, cells with supernumerary centrosomes that are unlinked and scattered over a larger area also generate a Golgi organization defect [47].

Cells with extra centrosomes were recently reported to have disrupted cell migration due to the presence of multiple scattered microtubule organizing centers [47]. Since it was suggested that the centrosomal linker effectively joins the two centrosomes into one functional MTOC [7], we investigate whether unlinked centrosomes would have the same effect on cell migration as supernumerary centrosomes. Random migration of RPE1 C-Nap1 KO cells was compared to RPE1 wt cells and revealed a 35–46% decrease in the velocity of C-Nap1 KO cells (Fig 6A and 6B). A decrease in cell migration speed of C-Nap1 KO cells was also observed in the directional migration using the wound healing assay. Thus, centrosomal linker deficient RPE1 cells migrate with reduced speed (Fig 6D and 6E). In contrast, the directionality of the random movement was unaffected by the absence of the centrosomal linker (Fig 6C). The exact nature and molecular connection of the reduced cell migration in C-Nap1 KO cells is presently unclear.

Taken together, the centrosomal linker is important for joining the two centrosomes into one functional MTOC unit during interphase. A relatively small increase in centrosomal distance already affects the organization of the Golgi and has consequences on cell migration. It will be interesting to see how this relatively modest defect in centrosomal linker deficient cells impacts upon the development and function of an organism.

## Materials and Methods

### Cell Lines and Treatments

HeLa and U2OS cells were cultured in Dulbecco's Modified Eagle's Medium (DMEM) Glutamax (Gibco) supplemented with heat inactivated 10% (v/v) FBS and 2 mM L-glutamine. hTERT-RPE1 cells were cultured in Dulbecco's Modified Eagle's Medium F-12 Nutrient mixture (DMEM/F-12, Gibco) supplemented with 10% FBS and 2 mM L-glutamine. All cell lines were cultured at 37°C in a humidified atmosphere with 5% CO_2_. Plasmid transfection was performed using Lipofectamine LTX according to manufacturers (Life Technologies) instructions. For siRNA-based experiments, Lipofectamine RNAi MAX was used according to manufacturers instructions. The following siRNA oligos were used: Non targeting siRNA (Human Dharmacon ON-Target plus, Nr. 1, Thermo Scientific, sequence: 5´-UGUUUACAUGUCGACUAA-3´) and C-Nap1 siRNA (Human CEP250 Dharmacon ON-TARGET plus, Nr. 1 and 3, Thermo Scientific, sequence: 5’-GAGCAGAGCUACAGCGAAU-3’ and 5´- AAGCUGACGUGGUGAAUAA-3). Microtubule depolymerization was performed using nocodazole at 100 nM or 5 μM for 1 h. Microtubule stabilization was performed with 10 μM taxol for 1 h. In order to depolymerize actin filaments, 1 μM Cytochalasin D was used for 1 h. Dynein was inhibited with 0.125 mg/ml Ciliobrevin D for 1 h.

### Targeted Gene Knockout Using ZFN and a Donor Vector

Sigma Aldrich designed a ZFN with a specific cut site in exon 15 of the CEP250 gene (coding for C-Nap1). The donor vector was constructed as reported [56] by PCR amplification of the genomic locus 800 bp upstream and downstream of the ZFN cut site using primers with the following sequences FW1: 5´-GCTGAGGCAGGAGAATCTCTTG-3´ and REV1: 5´-GGGCCAGCTGT CTGGCTGC-3´. The PCR product was subsequently subcloned in pJet 1.2 vector. The ZFN cut site was mutagenized in the donor vector by PCR mutagenesis. The 3xSTOP codon in every frame-Neomycin resistance cassette was inserted at the cut site. RPE1 wt cells were co-transfected with the ZFN plasmids and the donor vector using electroporation (Invitrogen, Neon transfection system). After transfection cells were cultured for 2 d at 37°C and then for 2 d at 30°C in order to increase ZFN efficiency. 4 days after the transfection, cells were seeded in 96-well plates (100 cells per well) in the presence of 0.5 mg/ml G418 for selection. 2–3 weeks after the selection onset, colonies were picked and screened for the presence of the Neomycin resistance gene at the correct genomic locus with primers FW2: 5´-TGCCTGTAATCCCAACTACTCG-3 and REV2 5´-TGTGCGAGG CCAGAGGCC-3´. The wt genomic locus was amplified using FW1 and REV1 primers. Absence of wt mRNA was confirmed by using FW3: 5´-CTGTGTGCAGCAGAATGGAGGCC-3 and REV3: 5´- CCTCTAGAGCCGCTTTCTCTCG-3´ primers that would amplify from wt exon 14 to exon 15, but could not amplify the mutated exon 15 probably because of its larger size.

### Immunofluorescence Microscopy

For indirect immunofluorescence, cells were fixed with ice-cold methanol for 5 min at -20°C or with 4% PFA for 15 min at room temperature. Cells were permeabilized with 0.1% Triton X-100 for 10 min, blocked with 10% (v/v) fetal calf serum (FCS) for 30 min and stained with antibodies in 3% (w/v) BSA (bovine serum albumin) in PBS. DNA was stained with Hoechst 33342 (0.2 g/ ml, Calbiochem). The following antibodies were used in immunofluorescence microscopy experiments: anti-C-Nap1 (BD Biosciences, recognizes N-terminus of C-Nap1), anti-C-Nap1 [16], anti-rootletin [16], anti-Cep68 (kind gift from E. Nigg [13]), anti-γ-tubulin (Abcam, TU-30), anti-Nek2 [16], anti-pericentrin (Abcam, ab-4448), anti-LRRC45 [12], anti-α-tubulin (Sigma, T-9026), anti-GM130 (Cell Signalling, D681), anti-MM491 (human centrosome auto-immune serum), anti-Cep164 (kind gift from G. Pereira [57], anti-Sas6 (Santa Cruz, sc-81431), anti-Cep135 (raised against a recombinant Cep135 fragment of amino acids 1–658 in rabbit by EuroGentec), anti-PCM-1 (kind gift from O. Gruss [34]), anti-acetylated-tubulin (C3B9 monoclonal antibody; kind gift of G. Pereira). Secondary antibodies were donkey anti-rabbit IgG coupled to Alexa Fluor 488, Alexa Fluor 594 or Alexa Fluor 647, donkey anti-mouse IgG coupled to Alexa Fluor 555 or Alexa Fluor 488, and donkey anti-goat IgG coupled to Alexa Fluor 555 (all 1:500; Invitrogen) and donkey anti-human coupled to Alexa Fluor 488 (used 1:200).

Imaging was performed on a DeltaVision RT system (Applied Precision) with an Olympus IX71 microscope equipped with FITC (fluorescein isothiocyanate), TRITC (tetramethyl rhodamine isothiocyanate) and Cy5 filters (Chroma Technology), a plan-Apo ×100 NA 1.4 and ×60 NA 1.4 oil immersion objective (Olympus), a CoolSNAP HQ camera (Photometrics), a temperature controller (Precision Control) and Softworx software (Applied Precision). Confocal imaging was performed using Zeiss LSM780 microscope with standard equipment.

Centrosome distance was manually calculated in 3 dimensions using the formula (3D distance)^2^ = (2D distance)^2^+(z stack distance)^2^.

### Live Cell Imaging

Live cell imaging was performed using the Nikon Biostation microscope IM-Q (cell migration assays) or DeltaVision RT system (fluorescence live cell imaging). Both microscopes were used at 37°C in a humidified atmosphere with 5% CO_2_. All live cell imaging experiments were performed with Ibidi glassware. Directionality index was calculated as a ratio between Euclidean distance and accumulated distance using the following formula: D = (Euc.dist.)/(acc.dist.). Wound healing assay migration speed was calculated as the average speed of the cell front.

### SDS-PAGE and Immunoblotting

Cells were collected by trypsinization. After washing with PBS, the cells were lysed in 10 mM Tris-Cl pH 7.5, 150 mM NaCl, 5 mM EDTA, 0.1% SDS, 1% Triton X-100, 1% deoxycholate supplemented with 1 mM PMSF (Sigma) and protease inhibitor cocktail (Roche) for 30 min cell. Lysates were centrifuged and the supernatant was boiled with Laemmli buffer. SDS-PAGE was performed as previously described [58]. Transfer to membrane was done using a BioRad Mini-Transblot Electrophoretic Transfer System. The membranes were subsequently blocked in 5% non-fat milk in TBS-T. The following primary antibodies were used: anti-C-Nap1 (BD Biosciences), anti-GAPDH (Cell Signalling Technology), anti-α-tubulin (Sigma, T-9026); with appropriate secondary antibodies: donkey HRP-coupled anti-mouse and donkey HRP-coupled anti- rabbit antibodies (from Jackson laboratories).

### Transmission Electron Microscopy

Cells were grown on coverslips and fixed using 2.5% glutaraldehyde in 0.1 M Na cacodylate buffer, pH 7.2, at room temperature for 30 min. The cells were subsequently washed with 0.1 M Na cacodylate buffer and postfixed with 2% osmium tetroxide in Na cacodylate buffer for 1 h on ice. The samples were washed and contrasted in 0.5% uranyl acetate over night. The samples were subsequently washed and gradually dehydrated by immersing them in a graded ethanol solution from 50, 70, to 90% and finally two times in 100% ethanol. Dehydrated cells were embedded in Epoxy medium using Epoxy Embedding kit (Fluka) and serial sections were generated using Reichert Ultracut S Microtome (Leica Instruments). Sections were post-stained with 2% uranyl acetate (in 70% methanol) and lead citrate. Finally, serial sections were viewed using a CM120 electron microscope (Phillips Electronics), operated at 120 kV, and images obtained by a Keen view CCD camera (Soft imaging systems).

### Image Processing and Analysis

ImageJ software was used for image analysis [59]. Centrosomes were counted as separated if the distance between them exceeded 2 μm. Mitotic cells were excluded from this analysis. For fluorescence intensity quantification of PCM-1, a square 3x3μm was used either having the centrosomal pair (in the case of wt) or having the mother or daughter centrosome in the center (separately, in the case of C-Nap1 KO clones). Average of background intensities were subtracted from each measurement in each channel. All statistical analyses were performed using GraphPad PRISM software.

## Supporting Information

S1 Fig Analysis of RPE1 C-Nap1 KO cells for centrosome localization of marker proteins. (A) Interphase RPE1 wt and RPE1 C-Nap1 KO (clone 7) cells were fixed and stained with the indicated antibodies. DNA was stained with DAPI. The inlets are 3-fold magnifications of the centrosomes in the main figure. Bars: 5 μm. (B) As in (A) but with RPE1 C-Nap1 KO (clone 17) cells. Bars on the smaller and larger images are 2 μm. (C) Normalized fluorescence intensity of PCM-1 signal in RPE1 wt cells with paired centrosomes (light blue). In RPE1 C-Nap1 KO cells, fluorescence intensity around the mother centriole that was stained with Cep164 (dark blue) and the daughter centriole (medium blue) was determined. The intensity distribution of N = 50 cells was analyzed for each cell type. Error bars are SEM.
(EPS)

S2 Fig EM analysis of centrioles from RPE1 and RPE1 C-Nap1 KO cells. Shown is a representative cross section through a centriole of RPE1 wt and RPE1 C-Nap1 KO cells. Both centrioles have the same structural appearance. Bars: 50 nm.
(EPS)

S3 Fig Cilia formation in RPE1 C-Nap1 KO cells. (A) RPE1 wt and RPE1 C-Nap1 KO cells were serum starved for 48 h to induce cilia formation. Cycling and serum starved cells were fixed and stained with the indicated antibodies. DNA was stained with DAPI. Bar: 5 μm. (B) RPE1 C-Nap1 KO cells form cilia as RPE1 wt cells. Cycling and serum starved cells from (A) were quantified for cilia formation. N = 40–60. Bars are SEM from three independent experiments.
(EPS)

S4 Fig RPE1 C-Nap1 KO cells do not have a mitotic defect. Mitotic RPE1 wt and RPE1 C-Nap1 KO cells were stained with anti-tubulin and anti-γ-tubulin antibodies. DNA was stained with DAPI. Cells were analyzed for spindle and chromosome missegregation defects. This analysis does not exclude a kinetic defect in spindle assembly in RPE1 C-Nap1 KO cells. Size bars: 5 μM.
(EPS)

S5 Fig Confirmation of actin depolymerization upon cytochalasin D treatment. RPE1 wt and RPE1 C-Nap1 KO clone 7 cells were incubated for 1 h with DMSO or Cytochalasin D. Fixed cells were stained with Phalloidin-Atto 565 and DAPI. Cells treated with Cytochalasin D do not have actin filaments.
(EPS)

S6 Fig Centrosome distance of C-Nap1 KO cells is not affected by dynein inhibition. (A) RPE1 wt and RPE1 C-Nap1 KO cells were treated with and without the dynein inhibitor ciliobrevin D. Fixed cells were analyzed with the indicated antibodies. GM130 staining was used as Golgi marker and anti- γ-tubulin staining as centrosome marker. DNA was stained with DAPI. Dispersal of the Golgi indicates that dynein was inhibited by ciliobrevin D. Bar: 10 μm. (B) Quantification of (A). N = 40–60 per experiment per condition. Error bars are SEM. Error bars are based on three independent experiments. We did not observe an increase in centrosome distance due to dynein inhibition. (C) RPE1 wt and RPE1 C-Nap1 KO cells were transfected with GFP or the dynein inhibitor p50-GFP. Fixed cells were analyzed with the indicated antibodies. DNA was stained with DAPI. Dispersal of the Golgi indicates that dynein was inhibited by p50-GFP. Bar: 10 μm. (D) Quantification of (C). N = 40–60 per experiment per condition. Error bars are SEM. Error bars are based on three independent experiments. We did not observe an increase in centrosome distance due to dynein inhibition.
(EPS)

S7 Fig Linker status in RPE1, U2OS and HeLa cells upon siRNA depletion of C-Nap1 and microtubule depolymerisation. (A) C-Nap1 of RPE1 cells was depleted by siRNA. A non-specific siRNA (NSC) was used as control. Depletion of C-Nap1 was shown by immunoblotting with anti-C-Nap1 antibodies. Tubulin was used as loading control. (B) C-Nap1 depleted RPE1 cells were incubated with and without 5 μM nocodazole for 1 h. Cells were fixed and centrosomes were stained with γ-tubulin. The centrosome distance of N = 80 cells per condition was determined; three independent experiments were performed. Shown is the centrosome distance of individual cells in a dot diagram. As for RPE1 C-Nap1 KO cells, we observed a synergistic effect of linker disruption and microtubule depolymerisation on centrosome distance. Error bars are SEM around the mean value of one representative experiment. (C) Cells of (B) were categorized according to centrosome distance. Centrosomes of a cell with a distance of >2 μm were counted as separated. Error bars are SEM around the mean value of three independent experiments. (D) As (A) but for U2OS cells. (E) As (B) but for U2OS cells. We observed a synergistic effect of linker disruption and microtubule depolymerisation on centrosome distance. (F) As (C) but for U2OS cells. (G) As (A) but for HeLa-ATCC cells. (H) As (B) but for HeLa-ATCC cells. HeLa-ATCC cells have a weak linker. Basal level of centrosome separation is already high. (I) As (C) but for HeLa-ATCC cells. (J) As (A) but for HeLa-B cells. (K) As (B) but for HeLa-B cells. The majority of HeLa-B cells do not have a functional centrosomal linker. Therefore, the basal separation of centrosomes is very high at 4 μm. (L) As (C) but for HeLa-B cells.
(EPS)

S8 Fig Linker morphology in U2OS and HeLa cells. (A) Linker morphology in U2OS cells. U2OS wt cells were incubated for 1 h with 5 μM nocodazole or the solvent control DMSO. Cells were fixed and stained with the indicated antibodies. Centrosomes with a distance of <2 μm were categorized as unseparated and with >2 μm as separated. Unseparated centrosomes had a functional linker. Separated centrosomes were associated with the linker proteins C-Nap1 and rootletin, however, rootletin failed to connect the two centrosomes. Note, short nocodazole treatment did not induce displacement of linker proteins in interphase cells. Bars: 2 μm. (B) Linker morphology in HeLa-ATCC cells. Treated and stained as in (A). Cells with unseparated centrosomes had a functional centrosomal linker. Bars: 2 μm. (C) Linker morphology in HeLa-B cells. Treated and stained as in (A). Even cells with unseparated centrosomes (<2 μm distance) did not have a functional centrosomal linker as indicated by the absence of connecting rootletin filaments. We cannot exclude low level of connection below our rootletin detection limit. Bars: 2 μm.
(EPS)
